# Development of the literacy scale for excellent traditional Chinese culture among college students

**DOI:** 10.3389/fpsyg.2026.1903193

**Published:** 2026-10-01

**Authors:** Xiumin Ouyang, Huixin Xiao

**Affiliations:** 1 School of Marxism, Sanming University, Sanming, China; 2 School of Marxism, Fujian Medical University, Fuzhou, China

**Keywords:** college students, excellent traditional Chinese culture, KAP framework, literacy, scale development

## Abstract

**Background:**

The Literacy scale for Excellent Traditional Chinese Culture (ETCC-LS) is an important measurement instrument for assessing college students’ cultural accomplishment and the effectiveness of university cultural education. Nevertheless, existing measurement tools show limited contextual suitability for Chinese college students. Based on the Knowledge-Attitude-Practice (KAP) framework, this study aimed to develop the ETCC-LS for the assessment of excellent traditional Chinese culture literacy among college students.

**Methods:**

Using a cross-sectional design with convenience sampling, an item pool was generated through literature review and expert consultation, then administered to college students across six universities in Fujian Province, China. Of 1,424 questionnaires distributed, 1,345 valid responses were retained and screened via item analysis before being randomly split into an exploratory subsample (*n* = 672, EFA) and a validation subsample (*n* = 673, CFA); internal consistency was assessed via Cronbach’s *α* and composite reliability.

**Results:**

The final ETCC-LS consisted of 11 items covering three dimensions: knowledge of traditional culture, attitude toward cultural openness, practice of cultural dissemination. The respecified three-factor model yielded acceptable fit: *χ*^2^/df = 4.869, RMSEA = 0.076, CFI = 0.984, TLI = 0.984. Standardized factor loadings ranged from 0.748 to 0.989. Cronbach’s *α* was high and stable across two subsamples (total scale *α* = 0.948–0.951), at or above the 0.95 redundancy risk threshold flagged for the knowledge of traditional culture and practice of cultural dissemination subscales. All dimensions met criteria for convergent validity (AVE > 0.50, CR > 0.70). Despite the high latent correlation (*r* = 0.893) between knowledge of traditional culture and practice of cultural dissemination, Fornell–Larcker criteria supported acceptable discriminant validity.

**Conclusion:**

The KAP-based ETCC-LS showed acceptable reliability and validity. It serves as a reliable measurement tool for assessing college students’ literacy of excellent traditional Chinese culture.

## 1 Introduction

Traditional Chinese civilization boasts a long history and profound ideological connotations, including spiritual virtues and philosophical wisdom such as patriotism, perseverance, self-discipline, ethical cultivation, and respect for teachers. It embodies the core spiritual pursuit and intrinsic cultural heritage of China (Lily et al., 2022). Under China’s national strategic plans, the inheritance and development of excellent traditional Chinese culture are regarded as the key steps toward building a culturally powerful socialist country and realizing the great rejuvenation of the Chinese nation (State Council of the People's Republic of China, 2017). “Excellent traditional Chinese culture” is an officially defined concept in Chinese cultural policy (Ding et al., 2024). It refers to positively valued heritage deemed worthy of inheritance, excluding the outdated or negative elements within traditional Chinese culture more broadly. College students act as a vital carrier for the inheritance and dissemination of Chinese culture and occupy a key position in sustaining cultural continuity. Previous research confirmed that systematic cultivation of student’s cultural identity can strengthen their national and political identity, regulate their daily behaviors and improve their mental health level (Zhu and Lin, 2022; Zheng, 2022). Relevant educational intervention studies further demonstrated that exposure to traditional cultural content can stimulate undergraduates’ innovative thinking and entrepreneurial competence (Geng et al., 2021).

The Knowledge-Attitude-Practice (KAP) measurement framework originated from the field of family planning and population studies in the 1950s (Launiala, 2009), and it has since been widely applied in multiple disciplines including education, public health, and cultural research. The core logic of this framework holds that knowledge lays the foundation for behavior outcomes, while attitude serves as the driving force shaping individual behaviors (Huang et al., 2023). Individuals can only form standardized, rational behavioral tendencies when they acquire systematic knowledge and develop positive attitudes (Cleary and Dowling, 2010). To date, abundant measurement tools following the KAP framework have been developed across diverse fields, including environmental literacy (Maleknia and Zamani, 2025), new media literacy (Lee et al., 2025), health literacy (Alqahtani et al., 2024), eHealth literacy (Yuan et al., 2023), physical literacy (Ma et al., 2024), digital literacy (Abou Hashish and Alnajjar, 2024), sustainable food literacy (Teng and Chueh, 2022), mental health literacy (Sibanda et al., 2022), and leisure literacy (Dai et al., 2019). Although KAP scales have been widely adopted in public health and media research, most existing instruments focus on non-cultural domains, and few are designed for measuring excellent traditional Chinese culture literacy. These tools therefore cannot be directly applied to assess such literacy among Chinese college students.

Scholars have put forward multiple definitions of cultural literacy from different theoretical perspectives in existing literature. Some scholars define it as the capacity to understand social identities, collective beliefs, and customs (Echeverri et al., 2020), while others define it as the ability to comprehend the histories, traditions, lifestyles, and ideologies of various cultural groups (Desmond et al., 2011). Instead of directly adopting a singular established definition of cultural literacy, this study synthesizes the common core connotations derived from the above scholarly interpretations and reconstructs a localized concept matching Chinese cultural contexts under the KAP framework. We conceptualize literacy of excellent traditional Chinese culture as a multidimensional construct covering college students’ knowledge of traditional Chinese culture, value judgments about the relative appeal of domestic traditional culture versus foreign popular culture, and active participation in cultural communication and practice. The KAP framework offers a theoretically coherent foundation for operationalizing cultural literacy, as it captures the progression from cultural knowledge to related attitudes and ultimately behavioral practice. Internationally, scholars have developed various assessment tools for cultural literacy, such as the cultural intelligence scale (Ang et al., 2007), measurement of cross-cultural communication behavior scale (Koester and Olebe, 1988), cultural identity scale (Pan et al., 2020), and cultural tolerance attitude scale (Stanley, 1996). However, most of these scales only measure single layer constructs such as cross-cultural adaptation or ethnic belonging; they fail to separate independent knowledge, attitudinal and behavioral dimensions as the KAP model requires, and cannot fully capture the complete logic of cultural literacy formation. Furthermore, most cross-cultural measurement tools are grounded in Western frameworks and therefore may not adequately reflect indigenous Chinese values (Ang et al., 2007; Pan et al., 2020), particularly the unity of knowledge and action.

We systematically retrieved Chinese and international databases, including CNKI, Wanfang Data, CBM, PubMed, Web of Science, Scopus, and CINAHL. Searching keywords covered cultural literacy, excellent traditional Chinese culture literacy, as well as knowledge, attitude, and practice related to traditional culture. The retrieval results showed that no psychometrically validated three-dimensional scale of excellent traditional Chinese culture literacy among college students has been developed yet. As the core talent reserve for the country’s future development, college students’ literacy of excellent traditional Chinese culture shapes their individual cultural identity and boosts the nation’s cultural soft power. Against the above research gaps, constructing a localized measurement tool based on Chinese traditional cultural values is of great theoretical and practical significance. In contrast to other instruments used to assess cultural identity, cultural intelligence, or cross-cultural adaptation, this study aims to develop a scale that captures culture-specific content embedded in traditional Chinese values, while differentiating three unified dimensions: knowledge, attitude, and practice. Thus, developing a psychometric scale for assessing college students’ literacy in excellent traditional Chinese culture based on the KAP theory would fill gaps in current research and offer theoretical and empirical references for global cross-cultural literacy studies from the perspective of Eastern civilization.

## 2 Methods

### 2.1 Design, sample, and setting

This study was conducted in two stages for scale development and validation. The first stage focused on item compilation and expert content validity evaluation. The second stage was formal data collection and systematic psychometric testing, including item analysis, EFA, CFA, reliability and validity verification. The sample size was determined strictly according to the classic factor analysis criteria. Stable factor extraction generally requires a participant-to-item ratio between 10:1 and 20:1, or an absolute valid sample size greater than 300 (Hogarty et al., 2005). A total of 1,424 questionnaires were distributed in this research, and 1,345 valid questionnaires were finally retained after removing invalid responses such as regular filling and missing values. With 20 formal scale items, the valid sample achieved a participant-to-item ratio of approximately 67:1, exceeding the rigorous threshold required for stable factor structure extraction and reliable CFA model estimation. Figure 1 outlines the overall study design and analytic sequence.

**Figure 1:**
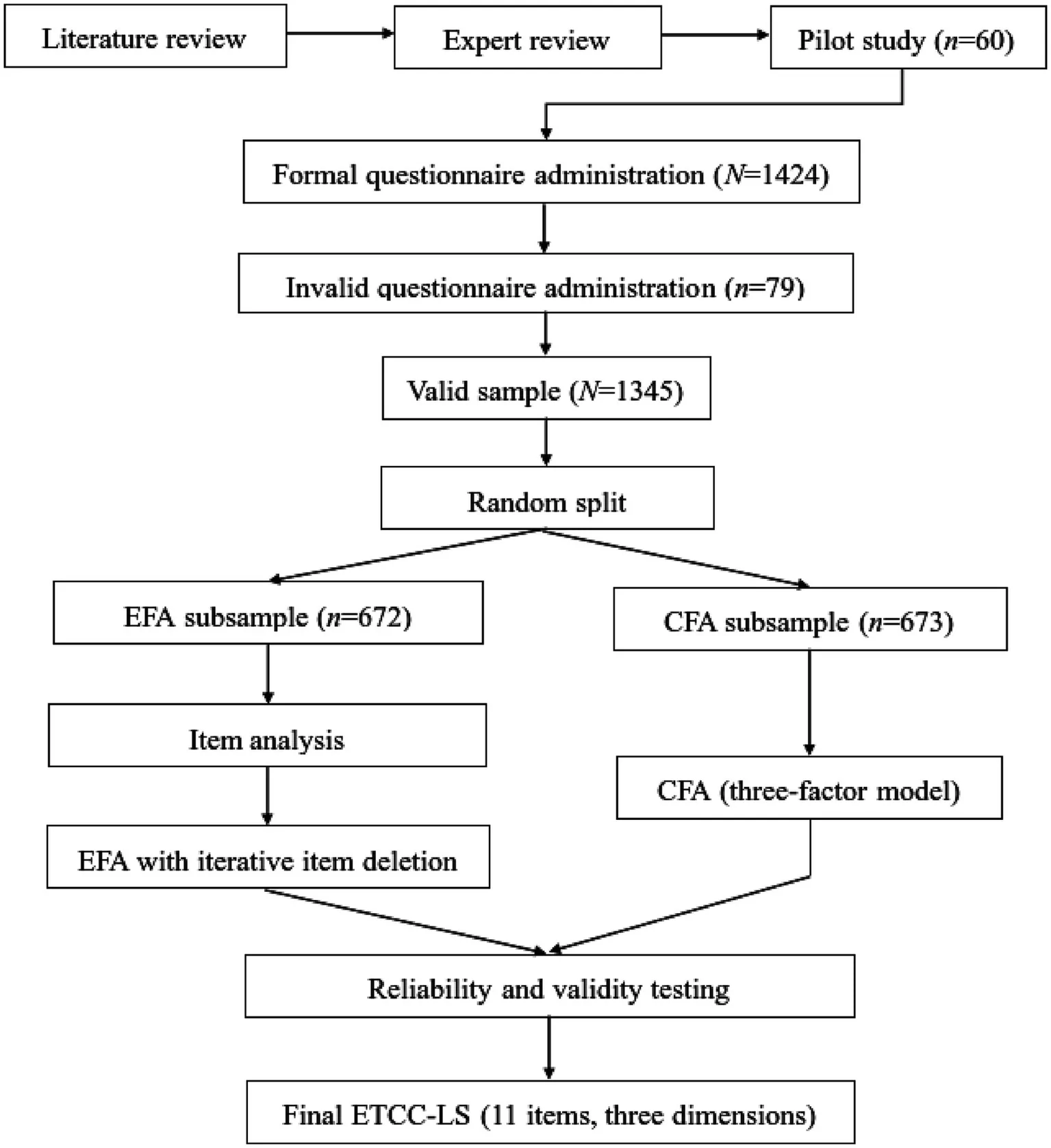
Study flowchart.

Convenience sampling was used to recruit college students from six universities located only in Fujian Province, a coastal province in eastern China. The inclusion criteria were as follows: (1) currently enrolled students without suspension or withdrawal records; (2) ability to understand scale items independently; and (3) voluntary participation.

### 2.2 Scale construction

#### 2.2.1 Formation of the research team

The research team consisted of one professor specializing in excellent traditional Chinese culture, one professor in social medicine, one expert in psychological measurement, and two postgraduate students majoring in traditional culture studies. All researchers received systematic training in psychometrics, EFA, and CFA before data analysis. Team members undertook differentiated and standardized tasks throughout the scale development process. Senior professors guided the theoretical framework construction, dimension division, and expert consultation procedures. The psychological measurement expert provided professional supervision of all statistical analyses, especially the implementation and interpretation of EFA and CFA. The two postgraduate students were mainly responsible for organizing expert review meetings, revising items based on expert feedback. The professor in social medicine was responsible for developing the initial item bank, conducting empirical analyses including item analysis, reliability test, EFA and CFA, to finalize the ETCC-LS scale.

#### 2.2.2 Item generation

Items for the ETCC-LS were developed through an iterative process. A systematic literature search was conducted across CNKI, Wanfang Data, CBM, PubMed, Web of Science, Scopus, and CINAHL for studies published between 2010 and 2025. After deduplication, approximately 268 unique records remained and informed the development of the initial item pool. Guided by the classic KAP framework, we developed items drawing on both domestic and international literature on traditional cultural cognition, cultural communication, and youth cultural values. This framework divides individual cultural responses into cognitive, attitudinal, and behavioral layers. We refined the number and wording of the items so that each item corresponded to one KAP dimension. After multiple rounds of revision and semantic polishing, we finalized a preliminary 24-item scale measuring college students’ literacy of Excellent Traditional Chinese Culture.

#### 2.2.3 Expert review

In order to ensure all items accurately reflected the core connotation of the research variables and aligned with the cognitive characteristics of college students, we conducted a qualitative open-ended expert review. It is important to clarify that this study adopted one-round qualitative expert consultation rather than the multi-round quantitative Delphi method. In accordance with Boateng et al. (2018), this open-ended expert review prioritized textual qualitative feedback instead of numerical scoring. Consequently, we did not compute quantitative indicators, including item-level content validity index (I-CVI), scale-level CVI (S-CVI), or Fleiss’ kappa for inter-rater agreement.

Five interdisciplinary experts were invited to the review panel, all satisfying three screening criteria: (1) expertise in research or practical experience in excellent traditional Chinese culture; (2) previous involvement in the development of psychometric scales; and (3) capacity to offer targeted, constructive revision suggestions. The panel consisted of two professors of education, one student affairs counselor with over 5 years of working experience, and two associate professors specializing in psychology. All experts received the complete draft of the scale with dimension definitions and a blank comment form 3 days ahead of the consultation meeting.

We collected expert comments covering four aspects: (1) whether each item corresponded to the theoretical connotation of its dimension, with no logical overlap or missing measurement content; (2) rationality of item allocation across dimensions and suggestions for item addition or deletion; (3) clarity of wording and suitability for college students; and (4) additional comments on dimension setting or item design. All verbal comments from the meeting were fully recorded, and handwritten comments from each expert were sorted and summarized.

All revision decisions followed unified consensus judgment rules (Herdman et al., 2002), with complete handling standards for all kinds of expert suggestions: (1) revisions supported by three or more experts were adopted directly; (2) revisions endorsed by two experts were adjusted after internal research team discussion based on the KAP framework; and (3) suggestions raised by only one expert were retained for theoretical re-evaluation after internal team discussion. Suggestions aligned with the core logic of this study’s KAP framework were partially integrated into the scale, while proposals conflicting with the conceptual definition of excellent traditional Chinese culture literacy were discarded with clear theoretical justifications recorded. For instance, we deleted the ambiguous item “I believe we should be continuing with our own orientation to spreading traditional Chinese culture” and optimized its wording to fit dimension definitions. After sorting all expert suggestions, two research members cross-checked all proposed revisions independently to avoid omission or subjective bias.

Four categories of adjustments were implemented according to expert opinions: (1) five items removed: Items 1, 4, 7, 10 and 14 (24-item draft) were deem inconsistent with core variable connotations by three experts. For example, Item 1 (24-item draft) “I am satisfied with my current inheritance of excellent traditional Chinese culture” was personal satisfaction rather than cultural identity and was therefore eliminated; (2) wording revision: five items were modified to fit the daily context of college students. For example, “I am willing to spend time improving myself through excellent traditional Chinese culture” was revised to “In daily life, I would cultivate my mind and body through traditional Chinese cultural activities (such as calligraphy, Chinese chess, etc.)”. (3) single dimension name adjusted: the original dimension “western cultural tendency” was renamed “cultural openness” based on feedback from two experts; and (4) one supplementary item added to fill measurement gaps within the practice of cultural dissemination dimension: “I enjoy using technologies such as the internet and new media to spread excellent traditional Chinese culture”, as pointed out by two panel members.

After expert review, the initial scale was revised a 20-item three-dimensional structure that was consistent with the theoretical framework and contextual adaptability. This interdisciplinary qualitative expert review procedure guaranteed the content validity of item pools and laid the foundation for the subsequent pilot test and formal psychometric validation. Item wording was further refined in the subsequent pilot study (see Section 2.2.4).

#### 2.2.4 Pilot study

Before formal data collection, we conducted a pilot study with 60 undergraduate students from Sanming University in Fujian Province to examine whether scale items were linguistically accurate, semantically unambiguous, and comprehensible for the target group (Ma et al., 2016). These 60 pilot participants were not included in the subsequent formal sample to avoid response bias. According to written feedback collected from pilot respondents, two items were revised to reflect college students’ media usage and optimize semantic expression: (1) Item 8 (20-item preliminary scale) was adjusted from “I will use Western popular culture to show my taste” to “I will use Western popular culture to express my personality”; (2) Item 17 (20-item preliminary scale) was revised from “I like to use the Internet to spread excellent traditional Chinese culture” to “I enjoy using technologies such as the internet and new media to spread excellent traditional Chinese culture.” Some participants pointed out that college students widely rely on new media channels to access cultural information, which prompted the revision of item 17. After completing these item adjustments, we carried out a formal large-scale cross-sectional questionnaire survey among undergraduates from several universities in Fujian Province. The formal survey aimed to assess the psychometric characteristics of the ETCC-LS, including internal consistency reliability and construct validity.

### 2.3 Data collection

#### 2.3.1 Data collection tools

Two types of research instruments were adopted for data collection. The first was a demographic questionnaire collecting participants’ background information, including gender, ethnicity, place of origin, academic year, discipline category, university type, and student cadre experience. The second instrument was the ETCC-LS scale composed of 20 items covering three dimensions: (1) knowledge of traditional culture (7 items); (2) attitude toward cultural openness (6 items); and (3) practice of cultural dissemination (7 items). Three items in attitude toward cultural openness were designed as reversed scoring items. All items were 5-point likert scoring ranging from 1 (strongly disagree) to 5 (strongly agree).

#### 2.3.2 Data collection process

Formal survey data were collected between September and October 2025. We adopted Wenjuanxing (a mainstream online questionnaire platform in China) as the data collection tool exclusively for offline classroom surveys. Researchers projected the questionnaire QR code onto classroom screens for students to scan and complete questionnaires on the spot. Before distribution, investigators informed participants of the research purpose, questionnaire contents and confidentiality agreements. Students could scan the QR code and take part in the survey voluntarily. To ensure data authenticity and prevent repeated filling, we set a restriction that each mobile device could submit responses only once. The questionnaire channel was opened during each offline survey period and closed immediately once participants finished their responses.

After all field investigations were completed, raw data were exported from Wenjuanxing platform. Two researchers independently screened the dataset to eliminate invalid questionnaires based on two criteria: (1) straight-lined questionnaires with identical answers to all items; (2) incomplete questionnaires with missing values on any item. A total of 1,424 questionnaires were distributed. Seventy-nine questionnaires were excluded, among which 51 were straight-lined responses and 28 contained incomplete missing value records. Ultimately, 1,345 valid samples were retained.

For subsequent psychometric testing, the full valid sample was randomly split at a 5:5 ratio into two mutually exclusive subsamples: the EFA subsample (*n* = 672) and the CFA subsample (*n* = 673). Random equal division is a concise random partitioning strategy suitable for large-sample research (Mondo et al., 2021). Scholars have confirmed that this splitting method performs well on datasets with high common variance, which facilitates the generation of balanced subsamples and stable factor structures for scale validation (Osborne and Fitzpatrick, 2012). All 1,345 total samples were used for item analysis (independent-samples t-test of extreme groups); the EFA subsample was used to explore the factor structure of the scale, and the CFA subsample was used to verify the convergent validity, discriminant validity and overall fit of the measurement model.

### 2.4 Statistical analysis

All statistical analyses were performed using SPSS 26.0 and AMOS 26.0. The validation sequence comprised item screening, KMO and Bartlett’s tests, EFA with varimax rotation, CFA, and internal consistency reliability analysis. Demographic variables were summarized with frequencies and percentages; no subgroup comparisons were conducted.

Item analysis was performed on the full sample (*N* = 1,345) before EFA and CFA. Three reverse-scored items (Items 5,7, and 8 of the 20-item preliminary scale) were recoded (revised score = 6-original score). Items with a corrected item-total correlation below 0.3 were eliminated (Nunnally and Bernstein, 1994). In a further check, independent-samples t-tests compared each item between the upper and lower 27% extreme groups defined by total score, and items showing no significant difference were removed.

Cronbach’s *α* coefficients were calculated on both the EFA and CFA subsamples for the final 11-item scale and its three subdimensions, with Cronbach’s *α* ≥ 0.70 considered acceptable (Taber, 2018); composite reliability (CR) was derived from the CFA subsample, with CR ≥ 0.70 as the criterion. Evaluating reliability on a subsample independent of the factor analyses avoids the inflated estimates that can arise when both are conducted on the same data. Preliminary item screening retained all items, so its impact on the independent subsample analyses was minimal.

For EFA, sampling adequecy was checked via the Kaiser-Meyer-Olkin (KMO) test and Bartlett’s test of sphericity. Principal component analysis with varimax rotation targeted three factor in line with the KAP framework. Items with factor loading lower than 0.5 on all factors or cross-loadings greater than 0.45 were deleted iteratively until a stable structure was obtained. Eigenvalue >1, cumulative explained variance >60%, and the scree plot were only treated as auxiliary references; although the third factor’s eigenvalue was <1, the three-factor structure was retained on theoretical grounds given the established KAP framework (Fabrigar et al., 1999).

CFA of the three-factor structure was conducted using maximum likelihood (ML) estimation. Model fit was evaluated with *χ*^2^/df, CFI, TLI, and RMSEA against a moderately lenient criterion (*χ*^2^/df < 5.0, CFI/TLI ≥ 0.90, RMSEA ≤0.08), as widely adopted in social science scale development. When fit was unsatisfactory, model modifications were guided by modification indices (>3.84) and restricted to changes consistent with the KAP framework.

Convergent validity was supported when the average variance extracted (AVE) of each dimension ≥0.5 and CR ≥ 0.70. Discriminant validity was established when the square root of each dimension’s AVE exceeded its correlations with the other latent factors (Fornell–Larcker criterion).

### 2.5 Ethics and research approval

This study was conducted in accordance with the Declaration of Helsinki and approved by the Biomedical Research Ethics Review Committee of Fujian Medical University (Approval No. 2025-481). Participants completed the anonymous questionnaire on site after scanning a projected QR code; an electronic informed consent statements covering research objectives, procedures, privacy protection, anonymity, and the right to withdraw at any time was displayed on the questionnaire homepage, and implied consent was deemed obtained upon voluntary completion. All participants were full-time undergraduate students who, under university enrollment regulations, were presumed capable of providing independent informed consent, so guardian consent was not required. No personally identifiable information was collected or stored.

## 3 Results

### 3.1 Demographic characteristics of the participants

Out of the 1,424 college students who were invited to participate in this study, 1,345 answered valid questions. The effective response rate was 94.5%. All the subsequent analyses were based on this valid sample of 1,345 participants. The participants’ demographic characteristics were as follows: 59.6% were female, 10.8% were minority, and 44.2% were freshmen. Among the academic background, 54.3% were science and engineering majors, and 55.8% attended local undergraduate universities. Among the socioeconomic background, 44.5% were rural, and 65.3% were student cadres. Demographic characteristics of the valid sample are shown in Table 1.

**Table 1:** Demographic characteristics of the participants (*N* = 1,345).

Variable	Characteristics	*n*	%
Gender	Male	543	40.37
	Female	802	59.63
Ethnicity	Han	1,200	89.22
	Ethnic minorities	145	10.78
Academic year	Freshman	595	44.24
	sophomore	313	23.27
	Junior	326	24.24
	Senior	111	8.25
Major category	Humanities and social sciences	302	22.45
	Science and engineering	731	54.35
	Physical education and sports	98	7.29
	Arts	214	15.91
University type	985/211 key universities	178	13.23
	Provincial key universities	417	31.00
	Local undergraduate universities	750	55.76
Student cadre experience	Yes	878	65.28
	No	467	34.72
Place of origin	Urban areas	388	28.85
	Towns and townships	358	26.62
	Rural areas	599	44.54

No missing data. The gender ratio was broadly reflective of the local university population.

### 3.2 Preliminary item analysis

A preliminary item analysis was performed on the 20-item ETCC-LS scale. Corrected item-total correlations for the 20 initial items ranged from 0.504 to 0.958 (Table 2), all exceeding the 0.3 threshold recommended by Nunnally and Bernstein (1994). According to the 27% extreme grouping principle, 363 participants were assigned to the high-score group and another 363 participants to the low-score group. The total score t-test verified significant inter-group differences (*t* = −42.736, *p* < 0.001), verifying the validity of grouping. The further t-tests for each individual item showed significant differences between high and low groups (*p* < 0.001), and the positive t values of the reversed items were consistent with the direction of reverse scoring. In summary, all 20 initial items passed the dual screening without elimination, and all items entered the subsequent EFA.

**Table 2:** Item-total statistics for the 20-item preliminary scale (*N* = 1,345).

Item	Scale mean if item deleted	Scale variance if item deleted	Corrected item-total correlation	Cronbach’s *α* if item deleted
Item 1	45.097	454.586	0.910	0.980
Item 2	45.051	454.258	0.936	0.979
Item 3	44.867	458.313	0.887	0.980
Item 4	44.631	471.855	0.791	0.981
Item 5	44.658	481.700	0.573	0.982
Item 6	44.819	461.995	0.892	0.980
Item 7	44.597	484.085	0.504	0.983
Item 8	44.610	485.399	0.512	0.983
Item 9	44.909	457.155	0.936	0.979
Item 10	44.823	459.260	0.939	0.979
Item 11	44.833	457.312	0.958	0.979
Item 12	44.548	469.699	0.839	0.980
Item 13	44.720	464.024	0.917	0.980
Item 14	44.825	458.820	0.950	0.979
Item 15	44.872	457.490	0.945	0.979
Item 16	44.642	467.745	0.877	0.980
Item 17	44.633	468.027	0.860	0.980
Item 18	44.710	466.185	0.856	0.980
Item 19	44.888	457.768	0.943	0.979
Item 20	44.528	470.508	0.814	0.981

No items were excluded at this preliminary item-analysis stage.

### 3.3 EFA results

EFA was conducted on the exploratory subsample (*n* = 672). For the initial 20 items, the KMO value was 0.972 and Bartlett’s sphericity test was significant (*χ*^2^ = 21,826.226, df = 190, *p* < 0.0001). Principal component analysis with varimax orthogonal rotation extracted three factors with a cumulative explained variance of 86.39%. The initial eigenvalues of the three factors were 15.165, 1.581, and 0.533, with the third factor’s eigenvalue below 1.

Items were then eliminated one by one according to the two statistical screening criteria (maximum factor loading <0.5 on all factors; absolute cross-loading ≥0.45 on two or more factors), and EFA was rerun iteratively after each deletion until a clean and stable factor structure without obvious cross-loading was obtained. Nine items (Items 4, 6, 9, 10, 11, 14, 15, 18, and 19 of the 20-item preliminary scale) were deleted due to low primary loading or severe cross-loading.

For the finalized 11-item scale, EFA yielded a KMO of 0.925 and a significant Bartlett’s test (*χ*^2^ = 28,818.755, df = 55, *p* < 0.0001). The Eigenvalues of the three extracted factors were 7.540, 1.503, and 0.477, and the cumulative explained variance reached 86.55% after rotation (Table 3). All retained items had factor loadings ranging from 0.715 to 0.873 (Table 4), each loading onto its corresponding factor without obvious cross-loading.

**Table 3:** Total variance explained of the scale (before and after item deletion).

Stage	Factor	Initial eigenvalues			Rotated sums of squared loadings		
		Amount (eigenvalue)	Variance %	Cumulative %	Amount (eigenvalue)	Variance %	Cumulative %
Original 20-items (Before deletion)	1	15.165	75.82	75.82	7.002	35.01	35.01
	2	1.581	7.91	83.73	6.909	34.55	69.56
	3	0.533	2.66	86.39	3.367	16.84	86.39
Final 11-items (After deletion)	1	7.540	68.55	68.55	4.558	41.43	41.43
	2	1.503	13.66	82.21	2.689	24.45	65.88
	3	0.477	4.34	86.55	2.273	20.66	86.55

Only the first three factors are listed.

**Table 4:** Rotated component matrixes (multiple rotations).

Item	Factor loading		
	practice of cultural dissemination	attitude toward cultural openness	knowledge of traditional culture
1. I enjoy using technologies such as the internet and new media to spread excellent traditional Chinese culture.	0.867		
2. In daily life, I would take the initiative to spread excellent traditional Chinese culture to my family, friends, and classmates.	0.864		
3. In daily life, I would cultivate my mind and body through traditional Chinese cultural activities (such as calligraphy, Chinese chess, etc.).	0.851		
4. I actively participate in diverse and varied traditional cultural education activities both inside and outside the classroom.	0.829		
5. In daily life, I obtain knowledge about traditional Chinese culture from new media and the internet, such as WeChat and Weibo.	0.795		
6. I would use Western pop culture to highlight my individuality.		0.873	
7. I think Western pop culture has greater international influence than traditional Chinese culture.		0.873	
8. I consider traditional Chinese culture to be cumbersome, difficult to learn, and unfashionable.		0.795	
9. I believe that traditional Chinese culture is extensive and profound, with significant value.			0.763
10. I take pride in traditional Chinese culture.			0.738
11. I am fully confident in the inheritance and development of excellent traditional Chinese culture.			0.715

① Items in this table are renumbered sequentially for the final 11-item scale after item deletion; ② The three reversed-scoring items (Table 3 Item 6, 7, 8) correspond to Items 5, 7, 8 in the original 20-item preliminary scale.

Based on the item content, the three factors were named as follows: (1) knowledge of traditional culture, reflecting college students’ cognition and understanding of core contents of excellent traditional Chinese culture; (2) attitude toward cultural openness, reflecting value judgments regarding the relative appeal of domestic traditional and foreign cultures; and (3) practice of cultural dissemination, reflecting college students’ proactive behaviors of inheriting, spreading and popularizing traditional culture in daily life.

### 3.4 CFA results

CFA was conducted on the validation subsample (*n* = 673). The initial three-factor model yielded sub-optimal fit: *χ*^2^/df = 6.232, RMSEA = 0.088, CFI = 0.977, TLI = 0.969. To improve model fit, modification indices were inspected, and the error terms of two item pairs within the same latent dimension (e4↔e5 and e2↔e3) were allowed to correlate. After residual correlation adjustments, the modified model yielded improved fit: *χ*^2^/df = 4.869, RMSEA = 0.076, CFI = 0.984, TLI = 0.984. In the respecified model (Figure 2), standardized factor loadings for all observed items ranged from 0.748 to 0.989, all statistically significant (*p* < 0.001). Before the three reversed items of the attitude toward cultural openness dimension were recoded, negative inter-factor correlations were observed. After recoding, all inter-factor correlations became positive. The latent-factor correlations were 0.893, 0.553, and 0.443.

**Figure 2:**
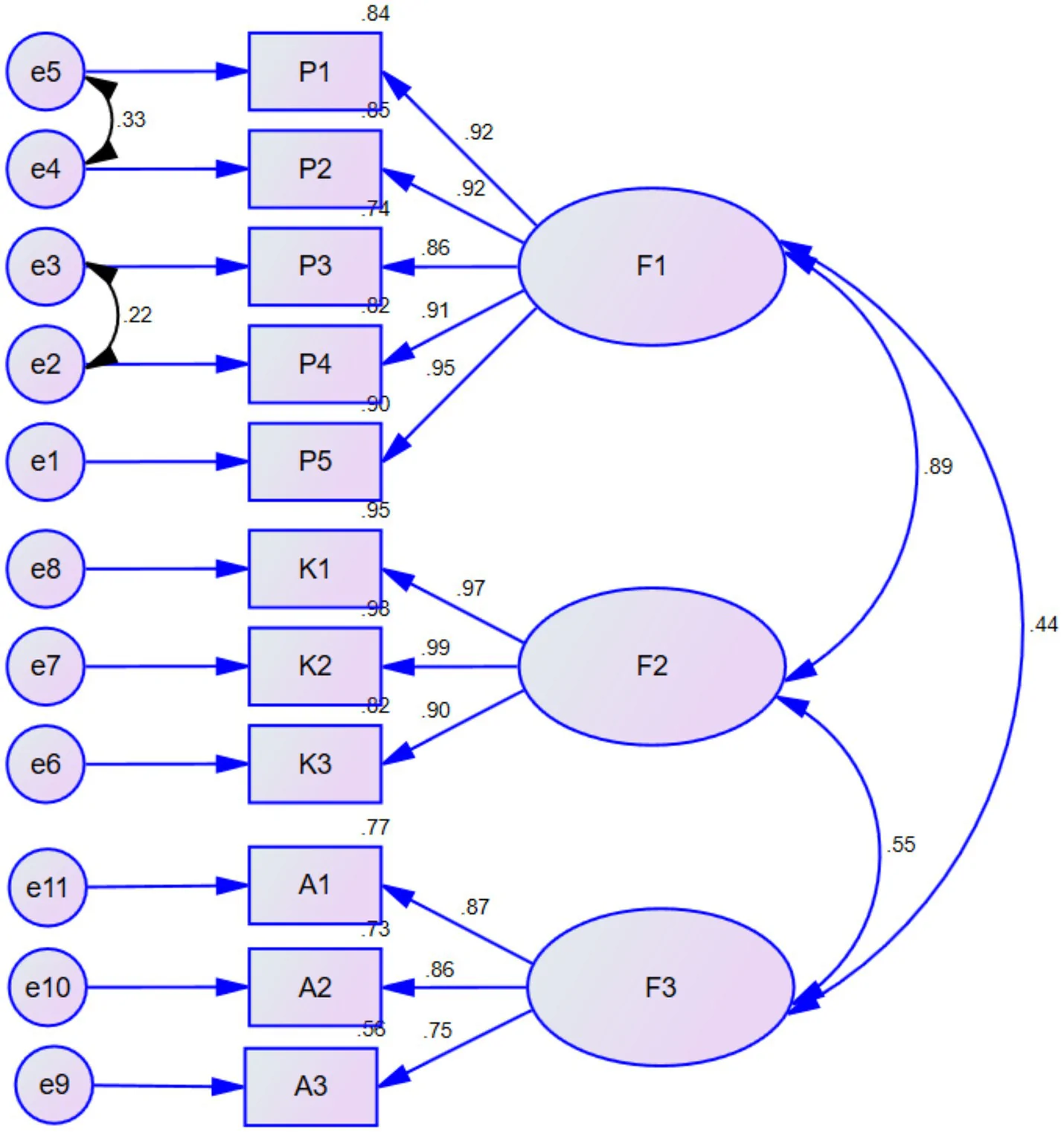
CFA model for the three-factor structure of the scale. F1 = practice of cultural dissemination, F2 = knowledge of traditional culture, F3 = attitude toward cultural openness.

Convergent validity was evaluated via standardized factor loadings, average variance extracted (AVE), and composite reliability (CR). As shown in Table 5, all standardized item loadings exceeded 0.70 (*p* < 0.001). The AVE values for practice of cultural dissemination (F1 = 0.831), knowledge of traditional culture (F2 = 0.915), and attitude toward cultural openness (F3 = 0.687) all exceeded 0.50. CR values ranged from 0.867 to 0.970, all above the acceptable criterion of 0.70. These outcomes supported satisfactory convergent validity. Discriminant validity was examined using the Fornell–Larcker criterion (Table 6). The square roots of AVE for the three factors were 0.912, 0.956, and 0.829, respectively, each exceeding the corresponding inter-factor correlations. The three-factor structure thus achieved acceptable discriminant validity.

**Table 5:** Convergent validity results.

Item			Standardized loading	AVE	CR
P1	←	F1	0.947^***^	0.831	0.961
P2	←	F1	0.907^***^		
P3	←	F1	0.861^***^		
P4	←	F1	0.922^***^		
P5	←	F1	0.919^***^		
K1	←	F2	0.973^***^	0.915	0.970
K2	←	F2	0.989^***^		
K3	←	F2	0.905^***^		
A1	←	F3	0.748^***^	0.687	0.867
A2	←	F3	0.857^***^		
A3	←	F3	0.875^***^		

^***^p < 0.001; F1 = practice of cultural dissemination, F2 = knowledge of traditional culture, F3 = attitude toward cultural openness.

**Table 6:** Discriminant validity assessment results.

Factor	practice of cultural dissemination	knowledge of traditional culture	attitude toward cultural openness
practice of cultural dissemination	**0.912**		
knowledge of traditional culture	0.893^***^	**0.956**	
attitude toward cultural openness	0.443^***^	0.553^***^	**0.829**

^***^p < 0.001; bold diagonal values represent the square root of AVE; off-diagonal values are latent factor correlation coefficients (Fornell and Larcker, 1981).

### 3.5 Reliability analysis results

Internal consistency was examined in both the exploratory (*n* = 672) and validation (*n* = 673) subsamples. In the exploratory subsample, Cronbach’s *α* was 0.951 for the overall 11-item ETCC-LS scale and 0.969, 0868 and 0.959 for the knowledge of traditional culture, attitude toward cultural openness, and practice of cultural dissemination dimensions, respectively. In the validation subsample, the total *α* was 0.948, and the corresponding dimensional values were 0.968, 0.863 and 0.964. All *α* values exceeded 0.70 in both subsamples (Nunnally and Bernstein, 1994). The highly consistent reliability across the two independent samples indicated stable internal consistency of the ETCC-LS.

Inter-item correlation matrices for each dimension using exploratory subsample (Table 7). Within the knowledge of traditional culture subscale, inter-item correlations ranged from 0.874 to 0.941 (mean = 0.913); within the practice of cultural dissemination subscale, from 0.776 to 0.888 (mean = 0.826); and within the attitude toward cultural openness subscale, from 0.643 to 0.749 (mean = 0.687).

**Table 7:** Subscale inter-item correlations and Cronbach’s *α* values (exploratory subsample, *N* = 672).

Subscale	Item	Correlation matrix (Item-level)	Cronbach’s *α*
Knowledge of traditional culture	K1-K2	0.941^**^	0.969
	K1-K3	0.874^**^	
	K2-K3	0.923^**^	
Attitude toward cultural openness	A1-A2	0.749^**^	0.868
	A1-A3	0.670^**^	
	A2-A3	0.643^**^	
Practice of cultural dissemination	P1-P2	0.888^**^	0.959
	P1-P3	0.810^**^	
	P1-P4	0.776^**^	
	P1-P5	0.863^**^	
	P2-P3	0.824^**^	
	P2-P4	0.814^**^	
	P2-P5	0.856^**^	
	P3-P4	0.779^**^	
	P3-P5	0.788^**^	
	P4-P5	0.836^**^	

^**^p < 0.01.

### 3.6 Final version of the scale

After thorough validity and reliability evaluation, the final version of the ETCC-LS comprised 11 items across three dimensions: practice of cultural dissemination (5 items), knowledge of traditional culture (3 items), and attitude toward cultural openness (3 items). All items adopt a 5-point likert scale, ranging from 1 (strongly disagree) to 5 (strongly agree). The three reverse-worded items belonging to the attitude toward cultural openness dimension were reverse-coded prior to the calculation of total and dimension scores to unify the response direction across all items. The total score of the ETCC-LS is computed as the sum of all 11 items, with higher scores indicating a higher level of excellent traditional Chinese cultural literacy. Dimension-level scores are computed as the average of item response within each corresponding dimension.

## 4 Discussion

Drawing on the KAP framework, this study developed and psychometrically validated the 11-item ETCC-LS for assessing college students’ excellent traditional Chinese culture literacy. The discussion examines the scale’s factor structure and other key psychometric outcomes, their theoretical implications, and the scale’s position relative to existing instruments.

### 4.1 Theoretical adaptability of the scale dimensions and structure

The final ETCC-LS yielded a stable three-factor structure derived from EFA and CFA: knowledge of traditional culture, attitude toward cultural openness, and practice of cultural dissemination. Rather than restating core assumptions of the KAP framework, the present section focuses on how empirical outcomes (factor loadings, inter-factor correlations, and retained item content) support or refine this theoretical structure for measuring excellent traditional Chinese culture literacy.

Compared with existing cultural related measurement instruments, the ETCC-LS targets literacy of excellent traditional Chinese culture instead of general intercultural competence or cultural identity attachment. Cultural intelligence scales assess adaptive capacity in multicultural contexts (Ang et al., 2007), and cultural identity scales focus on individuals’ sense of belonging to ethnic or national groups (Pan et al., 2020; Meca et al., 2023). By contrast, the ETCC-LS separates cognitive, attitudinal, and behavioral components, providing a domain-specific instrument for college students’ traditional culture literacy assessment.

Empirically, items within the knowledge of traditional culture dimension showed extremely high standardized factor loadings in CFA (0.905–0.989), reflecting strong homogeneity among the three retained items. Lynch (2020) treated cultural identity as a core cultural indicator, yet the present knowledge dimension differs conceptually. It captures cognitive comprehension and factual understanding of traditional culture content, rather than subjective identity attachment. This represents a meaningful improvement, because it disentangles cultural knowledge from identity-based belonging; thus, researchers can distinguish what individuals know about traditional culture from how strongly they identify with it. Consistent with KAP assumptions, knowledge constitutes the cognitive foundation for subsequent behavioral tendencies, which is further supported by the strong latent correlation between knowledge and practice observed in our data.

The attitude toward cultural openness dimension consists of three reverse-coded items with moderate factor loading (0.748–0.875). This dimension captures respondents’ value judgments toward the relative appeal of domestic traditional culture versus foreign popular culture. Here we acknowledge a conceptual tension with Parekh’s (2000) multicultural diversity thesis. Parekh (2000) advocated equal respect for all cultural traditions and openness toward cultural plurality. However, high scores on our dimension after reverse coding indicate endorsement of the value of excellent traditional Chinese culture, rather than a general willingness to embrace all foreign cultural inputs. In other words, this scale does not measure broad multicultural openness in Parekh’s sense; instead, it measures rational evaluative attitudes in regard to cultural alternatives. This is a limitation of the current item pool. Future revision may add items that directly tap respectful acceptance of cultural diversity to align more closely with Parekh’s theoretical idea.

Items retained in the practice of cultural dissemination dimension showed high factor loadings (0.829–0.947), covering both on-line and off-line interpersonal dissemination behaviors. Empirically, this behavioral dimension strongly correlated with the knowledge of traditional culture dimension (*r* = 0.893), suggesting that cognitive mastery of traditional culture is closely linked to self-reported dissemination behavior among college students in this sample. Taken together, the three-factor structure largely matches the hypothesized KAP based division, yet the high correlation between knowledge and practice suggests that these two components are not fully statistically independent in the present sample. After statistical item deletion, none of the retained items was judged semantically ambiguous or inconsistent with its target dimension definition, supporting the face validity of the final scale.

### 4.2 Reliability and validity analysis of the ETCC-LS

Across both exploratory and validation subsamples, the ETCC-LS demonstrated internal consistency coefficients satisfying conventional psychometric standards. Total-scale Cronbach’s *α* was 0.951 and 0.948 in the exploratory (*n* = 672) and validation (*n* = 673) subsamples, respectively; subscale *α* values in the validation subsample were 0.968 (knowledge of traditional culture), 0.964 (practice of cultural dissemination), and 0.863 (attitude toward cultural openness). Although these coefficients fall within ranges reported in existing cultural measurement literature (Jyoti and Kour, 2015; Pan et al., 2020), we do not interpret high *α* values as evidence of superior scale performance. As Streiner (2003) cautioned that Cronbach’s *α* above 0.95 frequently signals item redundancy, especially for short subscales. Because the total-scale *α* (0.948–0.951) also at or above the 0.95 threshold, the same redundancy caution should be applied when interpreting total scores. Indeed, high inter-item correlations within the knowledge of traditional culture and practice of cultural dissemination subscales (mean *r* = 0.913 and *r* = 0.826, respectively) confirm substantial item overlap. By comparison, the attitude toward cultural openness subscale showed moderate inter-item correlations and a more moderate *α* of 0.868. Users of the ETCC-LS should bear this redundancy risk in mind: high subscale reliability does not guarantee broad content coverage. Future revisions should focus on expanding the content coverage of knowledge of traditional culture and practice of cultural dissemination dimensions while preserving their conceptual boundary.

The high latent correlation between knowledge of traditional culture and practice of cultural dissemination (*r* = 0.893) was higher than Kline’s (2015) 0.85 warning threshold for discriminant validity risk. From the KAP theoretical perspective, cultural knowledge provides the cognitive basis for behavioral engagement, so a strong positive association is conceptually plausible. Even so, the Fornell–Larcker criterion was fulfilled: the square roots of AVE for the three dimensions ranged from 0.829 to 0.956, all larger than corresponding inter-factor correlations. Conceptually, knowledge of traditional culture and practice of cultural dissemination remain distinguishable constructs. However, the high empirical covariation implies that item refinement is still needed to improve between-dimension differentiation, and cross-sample replication is required to test whether this strong association persists in other populations.

EFA for the final 11-item scale yielded a cumulative rotated explained variance of 86.55%. Such a high value mainly stems from the generally high factor loadings of retained items. It should be emphasized that high explained variance does not mean complete statistical independence among dimensions; the substantial correlation between knowledge of traditional culture and practice of cultural dissemination illustrates that dimension level of covariation still exists despite theoretical guidance. During EFA, the third factor eigenvalue remained below 1.0, violating the Kaiser retention rule. In line with our analytical strategy, we retained the attitude toward cultural openness dimension grounded in KAP theoretical logic rather than applying mechanical statistical thresholds (Fabrigar et al., 1999). Future research could supplement additional items for this dimension to enrich its content coverage.

CFA results showed acceptable model fit: *χ*^2^/df = 4.869, RMSEA = 0.076, CFI = 0.984, TLI = 0.984. The *χ*^2^/df value met the lenient criterion prespecified in Section 2.4. CFI and TLI reached excellent level, whereas RMSEA = 0.076 sits near the upper acceptable boundary of the conventional ≤0.08 threshold (Hu and Bentler, 1999). By way of comparison, Hu et al. (2014) reported RMSEA = 0.04 for the Chinese Multiethnic Adolescent Cultural Identity Questionnaire, the relatively higher RMSEA in the present study may partly arise from residual correlations introduced for model respecification. Two residual correlations were introduced on content-based grounds: e4 (Item 1) and e5 (Item 2, both from the 11-item final scale) both address students’ willingness to disseminate traditional culture, one through daily interpersonal sharing and the other through online and new media, whereas, e2 (Item 4) and e3 (Item 3, both from the 11-item final scale) both concern involvement in cultural activities, one in educational settings and the other in self-cultivation practices (e.g., calligraphy, Chinese chess). Because these modifications were guided by modification indices, they should be regarded as exploratory, and the respecified structure warrants confirmation in independent samples. Convergent validity was supported: all standardized factor loadings exceeded 0.70, AVE > 0.50 and CR > 0.70 across dimensions. The Fornell–Larcker test further supported discriminant validity, with $\sqrt{\mathrm{AVE}}$ values for each factor exceeding pairwise latent correlations.

The pattern of latent factor correlations offers tentative insights into college students’ cultural psychological associations, which should be interpreted with caution given the cross-sectional nature of this study. The strong positive correlation between knowledge of traditional culture and practice of cultural dissemination (*r* = 0.893, *p* < 0.001) may reflect that richer traditional cultural cognition is associated with higher self-reported willingness for cultural dissemination. The moderate positive correlations between attitude toward cultural openness and the other two dimensions (*r* = 0.553, *r* = 0.443) may reflect that higher recognition of traditional culture value is associated with greater self-reported knowledge and dissemination engagement for traditional Chinese culture among college students. Notably, weak or differentiated correlations among the scale’s dimensions have also been reported in large-sample cross-cultural validation studies (Hadier et al., 2024). This pattern may represent a consistent empirical feature across cultural contexts. These observed associations do not prove causal effects. They may plausibly correspond to value preference patterns among participants in this sample; causal claims about cognitive effort allocation or intrinsic value conflict cannot be drawn from cross-sectional data.

## 5 Research limitations and future prospects

Several limitations of this paper should be noted, which also point to directions for further scale refinement and validation research.

First, geographical constraints exist in participant sampling. All participants were recruited from six universities in Fujian Province using convenience sampling. It should be noted that this sampling strategy carries two major limitations: (1) restricting data collection to a single provincial region may create regional cultural bias; (2) convenience sampling inherently weakens the representativeness of the population and generalizability of the findings. Future studies could adopt stratified multi-stage sampling covering universities across eastern, central and western China with different institutional types to test the cross-regional applicability of the ETCC-LS.

Second, some psychometric assessments were constrained by software functions and study design. Bootstrap confidence intervals for Cronbach’s *α*, composite reliability and model fit indices cannot be generated in SPSS and AMOS. Therefore, only point estimates are reported in this paper, without quantifying the uncertainty of these psychometric parameters. Besides, several important psychometric examinations were not conducted in the current research. We did not test measurement invariance across demographic subgroups including gender, ethnicity and academic major, so equivalence of scale performance among different student groups remains unconfirmed. Test–retest reliability data are also absent, leaving the temporal stability of scale scores unexamined. Furthermore, criterion validity evidence was not established. Future validation work should fill these gaps by implementing multi-group CFA for measurement invariance, collecting test–retest data to evaluate score stability, and introducing external criteria to verify criterion validity.

Third, some empirical weaknesses were observed in factor-structure results. In EFA, the third factor yielded an eigenvalue below 1.0, which deviates from the conventional Kaiser criterion. This factor was retained mainly based on KAP framework, and this empirical shortcoming should be acknowledged. The latent correlation between knowledge of traditional culture and practice of cultural dissemination reached *r* = 0.893, exceeding the 0.85 warning threshold for discriminant validity risk. Even though the Fornell–Larcker criterion was satisfied, such high covariation suggests partial conceptual overlap. The high inter-item correlations within these two subscales indicate strong item homogeneity, which may limit the construct coverage of the 11-item scale. Future scale development could enlarge the item bank to allow clearer conceptual separation between knowledge and practice indicators.

Finally, the content validity evaluation had inherent limitations. Only five scholars participated in the expert review. Since we adopted open-ended qualitative comments rather than standardized quantitative rating forms, quantitative indicators such as I-CVI, S-CVI, and Fleiss’ Kappa could not be calculated to quantify content validity and inter-expert agreement. Future research should invite a larger expert panel and use structured rating forms to obtain quantitative content validity evidence. Beyond the psychometric improvements mentioned above, researchers may also update practice dimension items to keep pace with emerging digital and AI-driven cultural communication forms. Long-term or intervention-based designs are also encouraged to explore dynamic changes in college students’ excellent traditional Chinese culture literacy.

## 6 Conclusion

The ETCC-LS was developed to measure college students’ literacy of excellent traditional Chinese culture from three interrelated dimensions: knowledge of traditional culture, attitude toward cultural openness, and practice of cultural dissemination. This scale offers a preliminary psychometric tool for universities to evaluate the effectiveness of excellent traditional Chinese culture education. It exhibits potential utility for subsequent intervention studies and can supply empirical reference for formulating relevant campus cultural education policies. Nevertheless, its generalizability should be treated with caution, as cross-regional applicability has not been fully verified. When applied in real-world educational scenarios, users need to take existing psychometric caveats of this scale into consideration.

## Data Availability

The raw data supporting the conclusions of this article will be made available by the authors, without undue reservation.

## Appendix A: Literacy scale for excellent traditional Chinese culture among college students (ETCC-LS)

**Table:**

Item	strongly disagree	relatively disagreed	uncertain	relatively agreed	strongly agree
1. I enjoy using technologies such as the internet and new media to spread excellent traditional Chinese culture.	1	2	3	4	5
2. In daily life, I would take the initiative to spread excellent traditional Chinese culture to my family, friends, and classmates.	1	2	3	4	5
3. In daily life, I would cultivate my mind and body through traditional Chinese cultural activities (such as calligraphy, Chinese chess, etc.).	1	2	3	4	5
4. I actively participate in diverse and varied traditional cultural education activities both inside and outside the classroom.	1	2	3	4	5
5. In daily life, I obtain knowledge about traditional Chinese culture from new media and the internet, such as WeChat and Weibo.	1	2	3	4	5
6. I would use Western pop culture to highlight my individuality.	1	2	3	4	5
7. I think Western pop culture has greater international influence than traditional Chinese culture.	1	2	3	4	5
8. I consider traditional Chinese culture to be cumbersome, difficult to learn, and unfashionable.	1	2	3	4	5
9. I believe that traditional Chinese culture is extensive and profound, with significant value.	1	2	3	4	5
10. I take pride in traditional Chinese culture.	1	2	3	4	5
11. I am fully confident in the inheritance and development of excellent traditional Chinese culture.	1	2	3	4	5
